# Setting Implementation Research Priorities to Reduce Preterm Births and Stillbirths at the Community Level

**DOI:** 10.1371/journal.pmed.1000380

**Published:** 2011-01-04

**Authors:** Asha George, Mark Young, Abhay Bang, Kit Yee Chan, Igor Rudan, Cesar G. Victora, Mickey Chopra, Craig Rubens, and the GAPPS expert group on community based strategies and constraints

**Affiliations:** 1 Health Section, UNICEF, New York, New York, United States of America; 2 Society for Education, Action and Research in Community Health, Gadchiroli, India; 3 Nossal Institute of Global Health, University of Melbourne, Melbourne, Australia; 4 Croatian Centre for Global Health, University of Split Medical School, Split, Croatia; 5 Centre of Population Health Sciences, The University of Edinburgh Medical School, Edinburgh, Scotland, United Kingdom; 6 University of Pelotas, Pelotas, Brazil; 7 Global Alliance to Prevent Prematurity and Stillbirth, Seattle Children's Hospital, Seattle, Washington, United States of America

## Abstract

Asha George and colleagues from the GAPPS group report the implementation research priorities to address prematurity and stillbirths at the community level that resulted from their recent expert consensus exercise.

**Summary Points**

- Preterm birth complications are the leading cause of neonatal mortality, contributing 1 million deaths annually. Stillbirths account for another 3.2 million deaths. Both causes of perinatal mortality are inextricably linked to maternal health and to conditions at birth.
- While some community-based interventions have proved effective in controlled settings and specific contexts, the implementation research challenge is to understand how to sustain these interventions at scale in different contexts.
- A systematic process based on the Child Health and Nutrition Research Initiative (CHNRI) methodology was used to score and rank implementation research questions regarding community-based maternal–newborn interventions that address prematurity and stillbirths in different contexts at scale.
- Of the 55 research questions that were reviewed in this way, the top five addressed equity (e.g., reaching the poor and marginalized, reducing financial barriers), behavioral practices and skills (e.g., engaging with social norms, identifying prematurity), and quality of care provided by community health workers. The top 15 questions encompassed issues pertaining to behavioral interventions, community health workers, referral, and managing health systems.

## Introduction

It is estimated that 3.2 million stillbirths occur each year globally, 1 million of which happen during birth [1]. In addition, complications from preterm birth (before 37 completed weeks of gestation) are the leading cause of death for newborns, contributing an additional 1 million or 12% of child deaths [2],[3]. In 2009, more than 200 stakeholders attended the International Conference on Prematurity and Stillbirth convened by the Global Alliance to Prevent Prematurity and Stillbirth (GAPPS, http://www.gapps.org/). The community expert group at the conference included 15 members drawn from technical and funding organizations in addition to program implementers and researchers from around the world (see Acknowledgments section for specific names). In their discussions, the group framed efforts to address preterm and stillbirths within the broader context of maternal–newborn interventions. As most of the evidence supporting these interventions emanates from research projects in controlled settings in specific contexts, the group identified the main challenge being implementing interventions at scale in different contexts. Based on these discussions, the group began a research prioritization exercise for implementation research on community-based maternal-newborn interventions that address prematurity and stillbirths at scale in different contexts. In this paper, we present the results of this exercise.

## Methods

A number of research prioritization efforts have recently been applied to various health topics and health system themes [4]–[7]. The GAPPS community expert group chose the methodology proposed by the Child Health and Nutrition Initiative (CHNRI) to systematically list and score research questions. The CHNRI methodology was selected because its conceptual framework [8]–[10] has been used in numerous areas by different national and international organizations [11]–[16] (further information on CHNRI methodology, validity, and potential limitations are discussed in Table S1). The group followed three main stages to derive research priorities (detailed in Box 1). Briefly, guided by the CHNRI methodology the group evaluated 55 research questions against five main criteria:

1. Is the research question answerable in an ethical way?
2. Does the research question have the potential to reduce the disease burden (due to prematurity and stillbirths)?
3. Is it likely that the proposed research would address obstacles to scaling up?
4. Would the proposed research attract funding support and national policy attention?
5. Would the research results be owned by local actors, including political authorities and elected representatives, health workers, district managers, and communities?

**Box 1. CHNRI Process**

**Stage 1: Defining the research context, questions, and criteria for priority setting**

When: May–September 2009

How: Group discussions and subsequent e-mails

Results:

- Consensus on research context defined by space (developing countries), time (the next 5–10 y), the population of interest (children under five years of age), and disease burden of interest (preterm and stillbirths). Respondents were also asked to keep in mind that all research questions started with the following introduction: “When implementing a community based maternal newborn intervention package that addresses prematurity and stillbirths in different contexts at scale…”
- Consensus around 55 implementation research questions grouped according to the following research domains: community engagement, behavioral skills and practices, community health workers, rational drug use, management health systems, and referral.
- Consensus on the five criteria used to rank the research questions: ethical answerability, disease burden reduction, ability to support scale-up, likelihood to attract financial and policy support, ownership by local actors.

**Stage 2: Enlisting experts to systematically score the research questions**

When: October 2009 – March 2010

How: Preliminary e-mails sent to 85 leading experts on community based approaches and maternal-newborn health in developing countries identified through a literature search and through snowballing of program managers. The spreadsheet was also translated into French and Spanish in order to ensure the participation of colleagues from Francophone Africa and Latin America.

Results:

- 42 experts agreed to participate
- 31 experts were able to complete the spreadsheets, independently scoring the 55 research questions by each of the five criteria by answering “Yes” (1 point), “No” (0 points), undecided (0.5 points), or insufficiently informed to answer the question (missing input).

**Stage 3: Computing and writing up results**

When: March–August 2010

How: An intermediate score was calculated for each of the five criteria and the RPS computed as the mean of all five intermediate priority scores [8]–[10] (Table S3). AEA scores were computed for each research question as the average proportion of scorers that agreed on the 55 questions asked (Table S1).

Results:

- 29 correctly completed spreadsheets analyzed with all 55 research questions systematically scored and ranked in order of priority and agreement.
- Draft circulated to all participants for feedback before being finalized.

Respondents were 39% women and diverse in terms of regional representation (26% sub-Saharan Africa, 16% Asia, 16% Latin America, 10% Europe, 32% North America). While a substantial number of respondents were based in North America, they all work full-time in developing country contexts. Half of the respondents were based in research institutions, whereas the other half were in charge of implementing programs whether through nongovernmental organizations, UNICEF country offices, or USAID headquarters. Nonrespondents were not significantly different from respondents (Table S2).

## Results

The research question that was highlighted as the most important out of all 55 reviewed was “Evaluate ways to reduce the financial barriers to facility births at the community level—e.g., user fee exemptions, emergency loans, conditional cash transfers, transportation vouchers, etc.” Other research questions among the top five prioritized also addressed equity issues (reaching the poor and marginalized), but also behavioral practices and skills (engaging with social norms, identifying prematurity) and service delivery (measuring and maintaining quality of care provided by community health workers). The remaining top ten research questions (Table 1) include other behavioral skills and practices (thermal care and feeding for preterm babies, birth planning), concerns about how to best motivate and compensate community health workers and their supervisors, and different dimensions of making referrals more effective. Congruent with the priority need to measure and maintain quality of care by community health workers, rational drug use by community health workers and community engagement with regard to audits was also listed among the top 25 research questions that received an overall research priority score (RPS) of 0.75 or greater (Table 2).

**Table 1:** The ten research questions that received the highest overall RPS.

Rank	Proposed Research Question	Answerable?	Burden Reduction?	Scale Up?	National Policy?	Ownership?	RPS	AEA
1	Evaluate ways to reduce the financial barriers to facility births at the community level (user fee exemptions, emergency loans, conditional cash transfers, transportation vouchers, etc)	0.930	0.663	0.845	0.877	0.895	0.858	0.821
2	Develop and validate strategies to identify preterm babies at community level by CHWs and family members	0.942	0.640	0.750	0.795	0.821	0.832	0.801
3	Evaluate different methods of behavior change that overcome harmful practices and promote positive cultural and social norms	0.904	0.696	0.909	0.886	0.772	0.829	0.794
4	Evaluate effective community-based strategies to reach the poor and marginalized	0.895	0.670	0.843	0.911	0.868	0.825	0.772
5	Evaluate ways to measure and maintain quality of care provided by CHWs	0.967	0.698	0.851	0.737	0.776	0.825	0.794
6	Evaluate ways to provide thermal care and feeding for the preterm baby	0.958	0.686	0.802	0.737	0.798	0.822	0.777
7	Evaluate financing measures at the community level that improve referral	0.915	0.500	0.848	0.729	0.877	0.817	0.779
8	Evaluate ways to motivate and compensate CHWs and their supervisors	0.983	0.596	0.929	0.700	0.817	0.814	0.785
9	Evaluate how to maximize referral compliance especially for the poor and marginalized	0.959	0.587	0.796	0.772	0.833	0.813	0.757
10	Evaluate ways to engage communities in birth planning for normal and at risk pregnancies	0.908	0.630	0.740	0.741	0.888	0.812	0.759

**Table 2:** Top 25 research questions by research area with a research priority score of 0.7 or above.

Rank	Research Area	Research Questions
12	Community engagement	Evaluate how community audits could improve access and quality of services
14		Evaluate how community engagement improves referral and counter-referral
2	Behavioral skills and practices	Develop and validate strategies to identify preterm babies at community level by CHWs and family members
3		Evaluate different methods of behavior change that overcome harmful practices and promote positive cultural and social norms
6		Evaluate ways to provide thermal care and feeding for the preterm baby
10		Evaluate ways to engage communities in birth planning for normal and at risk pregnancies
13		Assess the impact of initiation and continuation of Kangaroo Mother Care at home on survival of preterm/LBW babies in setting with high home births
15		Evaluate ways to ensure the sustained use of insecticide-treated bed nets by pregnant women and newborns
19		Evaluate ways to garner community support to ensure early and sustained breastfeeding
23		Evaluate ways to maintain CHW neonatal resuscitation skills
22	Rational drug use	Assess methods to ensure rational drug use among CHWs
5	Community health worker	Evaluate ways to measure and maintain quality of care provided by CHWs
8		Evaluate ways to motivate and compensate CHWs and their supervisors
16		Evaluate how CHWs can improve referral and counter-referral
17		Evaluate ways to assure continuous supply of essential medicines and inputs for CHWs
20		Evaluate ways to improve retention of CHWs
21		Evaluate how to measure good supervision for CHWs and different ways of providing it
24		Assess the optimal number of activities and population coverage required to maintain case load and skills of CHWs
25		Evaluate the equity impacts and effectiveness of CHW services when delivered with user fees or drug cost-recovery fees
1	Management and health systems	Evaluate ways to reduce the financial barriers to facility births at the community level (user fee exemptions, emergency loans, conditional cash transfers, transportation vouchers, etc)
4		Evaluate effective community-based strategies to reach the poor and marginalized
11		Evaluate demand-side financing mechanisms (e.g. insurance, demand side subsidies, vouchers)
7	Referral	Evaluate financing measures at the community level that improve referral
9		Evaluate how to maximize referral compliance especially for the poor and marginalized
18		Evaluate the barriers at the community and provider level that cause poor referral

Table 3 shows the ten research questions that were assigned the lowest RPSs. Several broad policy questions (human resource planning, gender profiles, budget flows, accountability, and monitoring systems) are listed here, along with some questions related to the sequencing of community interventions and one specific question regarding private provider practice (delayed cord clamping). Questions from almost all research avenues were found among the bottom ten research questions, suggesting that no one area was completely discriminated against by the scoring. Furthermore, even these lower-ranked research questions received relatively high RPSs compared to those arising from other CHNRI exercises. The RPS for all 55 questions ranged from 0.86 to 0.56, in contrast to other CHNRI exercises, which have generated RPS ranges from 0.90 to 0.25 [12]–[16]. This suggests that respondents collectively considered all implementation research questions as fairly important.

**Table 3:** The ten research questions that received the lowest overall RPS.

Rank	Proposed Research Question	Answerable?	Burden Reduction?	Scale Up?	National Policy?	Ownership?	RPS	AEA
46	Assess the gender distribution of CHWs and its implications in terms of their acceptability and effectiveness	0.925	0.343	0.602	0.619	0.633	0.639	0.574
47	Assess how CHWs and other kinds of frontline health workers are represented in human resource policies, strategies and legislation	0.925	0.271	0.556	0.583	0.692	0.638	0.593
48	Evaluate methods of integrating community-based data collection into district HMIS	0.930	0.298	0.636	0.526	0.579	0.628	0.565
49	Evaluate methods and levels of accountability that can be ensured	0.650	0.345	0.565	0.510	0.608	0.618	0.540
50	Assess the methods of tracking budget allocations and flow	0.889	0.256	0.482	0.636	0.651	0.611	0.548
51	Determine the minimum set of indicators required and the most effective monitoring system	0.825	0.298	0.609	0.535	0.544	0.608	0.547
52	Evaluate the sequencing and linking of different community level interventions	0.696	0.385	0.610	0.479	0.590	0.591	0.536
53	Evaluate different stages of community engagement (consultation, cooperation, co-learning, collective action), including their phasing, cost and effectiveness	0.816	0.267	0.663	0.453	0.548	0.587	0.518
54	Evaluate ways to ensure delayed cord clamping in deliveries assisted by private providers	0.933	0.278	0.478	0.343	0.616	0.573	0.532
55	Assess the optimal number of community groups that a community engagement facilitator can support	0.923	0.208	0.471	0.365	0.611	0.562	0.497

Research questions did vary in specificity. For example, broad questions such as “evaluate community-based strategies to reach the poor and marginalized” were scored alongside very specific questions like “evaluate ways to provide thermal care and feeding the preterm baby.” Both broad and specific questions were ranked in the top and bottom ten implementation research questions, suggesting that no bias existed against the kind of question asked.

The CHNRI methodology evaluates certain dimensions of each research question according to defined criteria. For example, “Evaluate methods and levels of accountability that can be ensured” was not considered to affect disease burden and “Evaluate ways to ensure delayed cord clamping in deliveries assisted by private providers” was not scored as likely to attract funding support or national policy attention. Among the five criteria, the most discriminative was the one related to disease burden reduction, while the least discriminative was the one regarding ethical answerability.

As mentioned, the relatively high mean scores assigned to questions across all criteria (apart from disease burden reduction) indicate that most of the respondents were fairly optimistic about the value of implementation research questions. Average expert agreement (AEA) ranged from 0.82 to 0.49. Similar to other CHNRI exercises, AEA showed a direct positive association with RPSs, indicating that there was more agreement among experts about what were the priority research questions. This is a property that is inherent to the way AEA is measured: very high or very low RPS scores require high levels of expert agreement, while substantial disagreement among experts will lead to RPS moving closer to a mean value [12]-[16].

To determine whether any systematic bias existed against certain questions due to the profile of the respondent, we analyzed scores for researchers and implementers separately. We found at least a 10% difference in the scoring assigned for 20% of the research questions (Table 4). The 11 questions for which there was a significant difference between researchers and implementers are spread across each research avenue, suggesting no one particular research area was affected by this difference of opinion. In ten out of these 11 questions, implementers ranked the implementation research question as being of higher value than researchers.

**Table 4:** Eleven research questions with a 10% or greater difference in RPS between implementers and researchers.

Rank	Proposed Research Question	Difference in Answerability Criterion	Difference in Burden Reduction Criterion	Difference in Scale-Up Criterion	Difference in National Policy Criterion	Difference in Ownership Criterion	Difference RPS	Difference AEA
37	Assess what communities consider as maternal-newborn health priorities and how communities compare maternal-newborn health with other development priorities	0.131	0.291	0.184	-0.030	0.099	0.135	0.650
22	Evaluate ways to improve retention of CHWs	−0.038	0.140	0.206	0.218	0.032	0.111	0.720
35	Evaluate different training approaches (including refresher training) for CHWs and their supervisors	−0.027	0.293	0.074	0.211	0.001	0.111	0.657
19	Evaluate ways to assure continuous supply of essential medicines and inputs for CHWs	0.083	0.265	0.162	0.135	0.028	0.135	0.751
36	Evaluate methods to prevent misuse of oxytocics	−0.101	−0.136	−0.092	−0.112	−0.112	−0.111	0.653
31	Determine culturally appropriate means to deliver skin to skin care (formative research of the cultural barriers, design of local solutions)	0.095	0.217	0.228	0.200	0.005	0.149	0.677
15	Assess the impact of initiation and continuation of Kangaroo Mother Care at home on survival of preterm/LBW babies in setting with high home births	0.022	0.105	0.237	0.154	−0.020	0.100	0.739
13	Evaluate demand-side financing mechanisms (e.g. insurance, demand side subsidies, vouchers)	0.106	0.167	0.165	0.152	−0.021	0.114	0.764
53	Determine the minimum set of indicators required and the most effective monitoring system	0.197	0.182	0.186	0.070	0.021	0.131	0.547
44	Measure the extent of household expenditures and their equity impacts	0.053	0.175	0.370	−0.010	0.137	0.145	0.609
51	Evaluate methods and levels of accountability that can be ensured	0.162	0.245	0.149	0.145	0.034	0.147	0.540

## Discussion

The top 25 research questions that have been prioritized span a broad range of issues (Table 2). These implementation research priorities address fostering and sustaining specific behavioral skills and practices at the community level, engaging communities in monitoring service delivery through audits, and improving referral. With regard to service delivery, a host of implementation research questions about the management of community health workers, along with the health system supports they require to function, were stressed. Finally, issues of equity, financing, and referral were highlighted, reflecting how community-based approaches cannot be dealt with in isolation from broader health system concerns.

While many of the implementation research priorities identified can be generalized across community-based maternal, newborn, and child health areas, a few distinctions may be particular to this specific exercise. Issues related to referral were present three times within the top 25 research questions. There is little implementation research on linking families from homes to facilities, or referral more broadly, in low-income countries [17]–[19]. While important gains have been made with task-shifting, effective and equitable referral remains vital, because the most serious cases of prematurity and other birth complications cannot be handled at the community level.

Implementation research questions related to community engagement and some other broader policy concerns central to managing health systems, such as human resource planning and monitoring systems, were overall not given high priority by respondents. Nonetheless, even the bottom ten research questions received high RPSs relative to other CHNRI exercises. This could be because the other exercises had more discriminatory criteria or because previous exercises compared different kinds of research (basic science versus implementation research). It may be easier for experts to discern between very different research areas (basic science versus implementation research) than to discern between areas of implementation research, which they may consider to be of relatively similar importance.

In addition, many of the implementation research questions do not by themselves contribute to improved maternal newborn outcomes. Their value comes forth when combined with other implementation issues that together make a more comprehensive and coherent community-based response with linkages to primary health care service delivery. It might therefore be difficult for respondents to think about specific implementation research questions in isolation from their broader social and health systems contexts.

The partiality toward some areas of implementation research could reflect the profile of respondents. A comparison of scoring by implementers and researchers did show some differences—not across any particular kind of research question, but in the direction of the bias, with implementers ranking implementation research questions higher than did researchers. The reasons for this difference among 20% of the questions are not known, but seem to indicate that implementers perceive the results of implementation research to be more powerful if effectively implemented.

While the CHNRI methodology provides a systematic and transparent way to rank research questions that purposefully avoids biases introduced by group dynamics dominated by powerful individuals, it still is a very lengthy process to undertake. Respondents must score 55 research questions according to five criteria that have three subcomponents each, resulting in 825 dimensions to respond to in the spreadsheet. This makes it a complex spreadsheet and likely does not help response rates. Eliciting participation via e-mail alone was not successful—only 42 out of 85 experts responded to the preliminary e-mail. The 42 experts that did express interest reflected a group that was more familiar with the GAPPS conference and had current working relationships with the lead authors who managed the exercise.

Despite these drawbacks, this exercise represents an important collaboration between researchers and program implementers to jointly identify the key implementation research questions vital to improving community-based maternal and newborn interventions that address preterm and stillbirths. The exercise also developed new criteria deemed more appropriate to implementation research, which require further testing and refinement to improve their discriminatory power.

Success in reducing stillbirth and prematurity rates, and in increasing the survival of preterm infants in low-income countries, is strongly dependent on achieving high and equitable coverage with existing cost-effective interventions [20],[21]. Yet coverage of such interventions remains unacceptably low in most countries. For example, across 68 countries with the highest mortality, only 54% of women deliver with a skilled birth attendant and 38% receive a postnatal visit [22]. Furthermore, coverage levels are particularly low among poor and rural families in these countries. Community-based interventions are therefore essential to reach population subgroups whose current access to health facilities is severely limited. The effect of expanding coverage of family and community care to 90% can by itself lead to a 15%–32% reduction in neonatal mortality [22]. Nonetheless, the knowledge gaps around how to sustain these programs at scale in different contexts remain significant.

## Conclusion

While important reviews [23]–[28] have helped to spur attention to community-based maternal newborn issues, with intriguing results for specific interventions [29],[30], the implementation research priorities identified in this article will, we hope, help to secure further research attention and financing for this important area. Priority research areas identified include equity concerns (such as removal of financial barriers and responsiveness to the poor and marginalized), specific behavioral skills and practices, and the management of community health workers including referral care. The challenge is now raised; will communities, governments, donors, research institutions, and international organizations respond?

## Supporting Information

Table S1 The CHNRI methodology for setting priorities in health research investments.
(0.04 MB DOC)

Table S2 Profile of respondents and non-respondents.
(0.04 MB DOC)

Table S3 All 55 implementation research questions scored and ranked.
(0.15 MB DOC)

## Abbreviations

AEA: average expert agreement
CHNRI: Child Health Nutrition Research Initiative
GAPPS: Global Alliance to Prevent Prematurity and Stillbirth
MDG: Millennium Development Goal
RPS: research priority score
WHO: World Health Organization
