# Supplementary material for: Setting Implementation Research Priorities to Reduce Preterm Births and Stillbirths at the Community Level
Source: PLoS Med. 2011 Jan 4;8(1):e1000380. doi: 10.1371/journal.pmed.1000380 (PMC3014929; doi:10.1371/journal.pmed.1000380)
Supplement: Table S1 — The CHNRI methodology for setting priorities in health research investments. (0.04 MB DOC) [file pmed.1000380.s001.doc]

**Table S1.** The CHNRI methodology for setting priorities in health research investments.

| **STAGE 1: Defining the context and criteria for priority setting**  Specifying the context a priori is a critical part of the CHNRI process, because priority scores for many research investment options may change substantially according to different contexts. According to CHNRI guidelines (**10**), the implementation research context was defined by space (developing countries), time (the next 5-10 years), the population of interest (children under five years of age) and disease burden of interest (preterm births and stillbirths). Respondents were asked to in particular keep in mind that all research questions were framed by the following introductory phrase: “*When implementing a community based maternal newborn intervention package that addresses prematurity and stillbirths in different contexts at scale…*”.  The core working group also adapted the five criteria to be used to score the research questions. The standard version of CHNRI methodology uses the set of five criteria, but they can be modified or changed in different contexts – in this case, to fit the context of the implementation research. The working group agreed to retain two of the standard CHNRI criteria: (i) answerability of the research question in an ethical way; and (ii) the potential of proposed research to reduce the existing disease burden (due to prematurity and stillbirths). Three standard CHNRI criteria were discarded: (iii) likelihood of effectiveness; (iv) likelihood of deliverability; and (v) predicted impact on equity. The group felt that those three criteria, as defined in standard CHNRI framework, were not sufficiently discriminatory or appropriate for implementation research questions for community based approaches. Instead, three new criteria were developed to replace them: (iii) likelihood that the proposed research would address program gaps for scaling up; (iv) likelihood to attract funding support and national policy attention; and (v) likelihood that the research results would be owned by local actors, including political authorities and elected representatives, health workers, district managers and communities. All community CHNRI group members then validated the criteria and finalized 55 research questions. |
| --- |
| **STAGE 2: Choice of technical experts, systematic listing and scoring of research investment options**  The first task of the core group of technical experts was to propose a large spectrum of research questions in a systematic way, according to the framework developed by CHNRI (supplementary **Tables S3, S4**). The conceptual framework for this process is described in detail elsewhere (**8-11**).  While a list of primarily biomedical research questions had been proposed to the group at the Seattle conference, the group felt that vital health systems concerns pertinent to how interventions are operationalised, like the management of community health workers and health systems supports for community level interventions, were not adequately reflected. We kept some of the behavioural research questions and in addition proposed new implementation research questions that fit within the following research avenues that were deemed of critical interest: (i) community engagement, (ii) community health workers, (iii) rational drug use, (iv) community level interventions (primarily behavioural), (v) referral and (vi) management/ health systems issues. The final list of 55 research questions were reviewed for potential gaps and finalized by the expert group through face to face consultations and over e-mail.  The finalized CHNRI score spreadsheet was then sent via email to 85 leading experts on community based approaches and maternal-newborn health in developing countries. They were identified through a preliminary literature search and through snowballing of program managers. Every effort was made to invite a mix of people with different backgrounds (clinicians, epidemiologists, public health experts, and programme managers ) and from different countries (both developed and developing ones), so that the mix contains a diversity of views. While the exercise was on research priorities, because it focused on implementation research, special efforts were made to include the perspectives of those in charge of implementing programs. The exercise was translated into French and Spanish in order to ensure the participation of colleagues from Francophone Africa and Latin America. Out of 85 experts engaged in research and implementation of maternal newborn programs, 42 expressed interest in contributing to this research prioritization exercise. The profile of responders and non-responders is presented in the **Supplementary Table S2**.  The co-ordinator of the project for GAPPS (AG) then invited the 42 experts with interest in implementation research to submit their scores. Every expert scored all 5 criteria, thus limiting potential impact of any single expert on overall scores. Out of 42 experts who initially agreed to take part in the exercise, 31 responded with a full list of research questions scored according to CHNRI criteria (74% response rate). Two spreadsheets had to be discarded due to errors in filling out the spreadsheet, so a total of 29 responses were analyzed. Respondents were fairly even in terms of gender (39% women) and diverse in terms of regional representation (26% sub-Saharan Africa, 16% Asia, 16% Latin America, 10% Europe, 32% North America). While a substantial number of respondents were based in North America, they were all engaged full-time on working in developing country contexts. Half of respondents were based in academic research institutions, whereas the other half represented those in charge of implementing programs whether through non-governmental organizations, UNICEF country offices or USAID headquarters. Non-respondents were not significantly different from respondents (see **Supplementary Table 2**). The process was conducted and completed via e-mail between October 2009 and March 2010. Further information on methods related to this part of the priority-setting process were presented elsewhere in greater detail (**17-19**). |
| **STAGE 3: Computations of “research priority scores”**  All the experts answered the questions listed in Box 1 by ‘Yes’ (1 point) or ‘No’ (0 points). They were also allowed to declare an informed but undecided answer (0.5 points) or declare themselves insufficiently informed to answer the question (missing input). Thus, the proposed research questions got a score for each of the five criteria as “the proportion of maximum possible points scored when an answer was given” (i.e., excluding the missing input). Each of the 55 listed research questions received five intermediate scores (each ranging between 0-100%), which were then multiplied by 100. CHNRI methodology allows for weighting of the intermediate scores based on the input from the external group of stakeholders. However, in this exercise the weights were not applied because it was not possible to appropriately define a relevant group of stakeholders for all contexts to which this exercise could potentially apply. The overall research priority score (RPS) was then computed as the unweighted mean of all five intermediate priority scores. The exact scores given to all 55 research questions from individual experts are presented in supplementary **Table S3**. The final list of priorities with intermediate and final priority scores for all 55 proposed research questions is presented in supplementary **Table S4**.  **Assessment of agreement between scorers**  CHNRI methodology has the ability to expose the issues of greatest agreement and controversy. This allows more focused discussion among experts following this exercise, and informs the investors and policy makers about the amount of controversy that surrounds each research question. The datasets that CHNRI methodology produces are not appropriate for application of the usual Kappa agreement statistics as discussed in detail elsewhere [**18,19**].  For each evaluated research investment option, AEA is informing us, for an average question, what proportion of scorers gave the same most frequent answer. This parameter accounts for missing answers, is unaffected by responses of ‘undecided’, and is also unaffected by the varying number of scorers per criterion and differences in scorer composition for the different criteria. |
| **Advantages and limitations of the CHNRI methodology**  The applied CHNRI methodology allows for the systematic listing and scoring of a large number of specific research questions. Other advantages of the CHNRI process include its well defined (a priori) context and criteria chosen for discriminating between research investment options, a highly structured way in which relevant information is obtained from the scorers, independent scoring that limits influence of strong-minded individuals on the rest of the scorers, and ability to expose points of greatest agreement and controversy.  Although the advantages mentioned above represent attempts to deal with many issues inherent to research priority setting, there are still some potential biases. One of them is related to the fact many possible good ideas (“research investment options”) may not have been included in the initial list of research options that was scored by the experts, and to the potential bias towards items that get the greatest press coverage. The spectrum of research investment options listed initially in this exercise was derived through a systematic process, but it is not endless and it cannot ever cover every single research idea. Therefore, the CHNRI process aims to achieve reasonable coverage of the spectrum of possible ideas. After the completion of the exercise, approximate scores and ranks for some specific research questions that are missing in the initial systematic list could still be estimated – either by relating them to the most similar questions on the list or by having those missed questions scored by a single expert (or by a group), using the CHNRI framework and then comparing the computed score to all other scores received for the originally listed research options.  Another concern over the CHNRI process is that its end product represents a possibly biased opinion of the involved group. We tried to balance the group with experts tasked with both research and implementation responsibilities. |
| **Validation of CHNRI methodology**  CHNRI methodology combines two ideas:  (i)“Principal component analysis” – a statistical technique which reduces a very complex system of large number of variables to a small number of relatively independent “principal components” which still capture a sizeable proportion of variation in the system; by defining a set of 5 “criteria”, CHNRI process effectively reduces a notoriously complex and multi-dimensional task of priority setting, which could be approached through an almost infinite number of “lenses”, into an exercise where the 5 most important (and reasonably independent) criteria for priority setting are clearly defined. They can even be weighted afterwards, in order of their importance to the users.  (ii) “Wisdom of the crowds” – this refers to the process of taking into account the collective opinion of a group of individuals rather than a single expert (or small number of experts) to answer a question, because it has been shown that the average of collective guesses are nearly always closer to the truth than any expert judgement. The pre-requisites for this process to work are: (i) Diversity of opinion (each person should have private information); (ii) Independence (people’s opinions aren’t determined by the opinions of those around them); (iii) Decentralization (people are able to specialize and draw on local knowledge); and (iv) Aggregation (some mechanism exists for turning private judgments into a collective decision – in this case, the CHNRI method).  The validation of CHNRI method based on the exercises conducted to date showed: (i) stability (correlation coefficients of over 90%) of scores given to same questions by the same experts in different points in time; (ii) almost identical scores of the same question scored by a larger group multiple times (score always falls within +1.7 points on a scale 0-100); and (iii) Monte Carlo simulations in random sub-samples of the larger group of scorers showed that the probability that the outcomes of the exercise could be substantially different if another group of experts conducted the scoring becomes incredibly small as soon as each criterion is scored by more than 17-23 rational persons with some knowledge of the problem; (iv) change of the context of the exercise leads the same group of experts to assign significantly different scores to the same research questions.  In comparison to other methods for setting priorities, in “expert panel”-type processes one very loud vote has a potential to heavily bias the process. During the GAPPS conference nine working groups defined priorities using Delphi-type processes, while three working groups used the CHNRI method. At the end of the conference, the rapporteurs from Delphi groups realised that it is not possible to have a discussion on all possible research options and keep in mind all their pros and cons all the time. Eventually, the group leaders ended up forwarding the ideas which they originally brought to the table and gained support for them from the rest of the group. This did not happen in the CHNRI group. |
