# Supplementary material for: Setting Implementation Research Priorities to Reduce Preterm Births and Stillbirths at the Community Level
Source: PLoS Med. 2011 Jan 4;8(1):e1000380. doi: 10.1371/journal.pmed.1000380 (PMC3014929; doi:10.1371/journal.pmed.1000380)
Supplement: Table S2 — Profile of respondents and non-respondents. (0.04 MB DOC) [file pmed.1000380.s002.doc]

**Table S2:** Profile of respondents and non-respondents

|  | | Gender | | Region | | | | | Organisational Base | | Total |
| --- | --- | --- | --- | --- | --- | --- | --- | --- | --- | --- | --- |
|  | | Women | Men | Sub-Saharan Africa | Asia | Latin America | Europe | USA | Research (University, WHO) | Implementing NGO, UNICEF, USAID |  |
| Respondents | Numbers | 12 | 19 | 8 | 5 | 4 | 3 | 11 | 15 | 16 | 31 |
| Non-respondents | Numbers | 3 | 8 | 4 | 2 | 0 | 1 | 4 | 2 | 9 | 11 |
| Total | | 15 | 27 | 12 | 7 | 4 | 4 | 15 | 17 | 25 | 42 |

Countries where respondents were based:

Bangladesh, India, Pakistan, Thailand

Burundi, Ethiopia, Niger, Malawi, Senegal, Zambia

Brazil, Argentina, Columbia

Sweden, Switzerland, UK, USA

Countries were non-respondents were based:

India, Nepal

Burkina Faso, Senegal, Kenya, Uganda

Denmark, USA
