# Supplementary material for: Setting Implementation Research Priorities to Reduce Preterm Births and Stillbirths at the Community Level
Source: PLoS Med. 2011 Jan 4;8(1):e1000380. doi: 10.1371/journal.pmed.1000380 (PMC3014929; doi:10.1371/journal.pmed.1000380)
Supplement: Table S3 — All 55 implementation research questions scored and ranked. (0.15 MB DOC) [file pmed.1000380.s003.doc]

**Table S3:** All 55 Implementation Research Questions Scored and Ranked

| **RANK** | **RESEARCH OPTION** | Answerable? | Burden reduct? | Scale-up? | Nat'l policy? | Ownership? | RPS | Difference implementers vs. researchers |
| --- | --- | --- | --- | --- | --- | --- | --- | --- |
| 1 | Evaluate ways to reduce the financial barriers to facility births at the community level (user fee exemptions, emergency loans, conditional cash transfers, transportation vouchers, etc) | 0.930 | 0.663 | 0.845 | 0.877 | 0.895 | 0.858 | 0.135 |
| 2 | Develop and validate strategies to identify preterm babies at community level by CHWs and family members | 0.942 | 0.640 | 0.750 | 0.795 | 0.821 | 0.832 | -0.040 |
| 3 | Evaluate different methods of behaviour change that overcome harmful practices and promote positive cultural and social norms | 0.904 | 0.696 | 0.909 | 0.886 | 0.772 | 0.829 | -0.065 |
| 4 | Evaluate effective community-based strategies to reach the poor and marginalized | 0.895 | 0.670 | 0.843 | 0.911 | 0.868 | 0.825 | 0.097 |
| 5 | Evaluate ways to measure and maintain quality of care provided by CHWs | 0.967 | 0.698 | 0.851 | 0.737 | 0.776 | 0.825 | -0.050 |
| 6 | Evaluate ways to provide thermal care and feeding for the preterm baby | 0.958 | 0.686 | 0.802 | 0.737 | 0.798 | 0.822 | 0.041 |
| 7 | Evaluate financing measures at the community level that improve referral | 0.915 | 0.500 | 0.848 | 0.729 | 0.877 | 0.817 | 0.064 |
| 8 | Evaluate ways to motivate and compensate CHWs and their supervisors | 0.983 | 0.596 | 0.929 | 0.700 | 0.817 | 0.814 | -0.062 |
| 9 | Evaluate how to maximize referral compliance especially for the poor and marginalized | 0.959 | 0.587 | 0.796 | 0.772 | 0.833 | 0.813 | 0.093 |
| 10 | Evaluate ways to engage communities in birth planning for normal and at risk pregnancies | 0.908 | 0.630 | 0.740 | 0.741 | 0.888 | 0.812 | 0.069 |
| 11 | Evaluate demand-side financing mechanisms (e.g. insurance, demand side subsidies, vouchers) | 0.895 | 0.512 | 0.840 | 0.886 | 0.851 | 0.805 | 0.073 |
| 12 | Evaluate how community audits could improve access and quality of services | 0.936 | 0.534 | 0.821 | 0.731 | 0.768 | 0.804 | 0.025 |
| 13 | Assess the impact of initiation and continuation of Kangaroo Mother Care at home on survival of preterm/LBW babies in setting with high home births | 0.907 | 0.660 | 0.683 | 0.806 | 0.694 | 0.801 | 0.025 |
| 14 | Evaluate how community engagement improves referral and counter-referral | 0.925 | 0.510 | 0.891 | 0.678 | 0.746 | 0.797 | -0.019 |
| 15 | Evaluate ways to ensure the sustained use of ITNs by pregnant women and newborns | 0.974 | 0.533 | 0.765 | 0.809 | 0.786 | 0.796 | 0.002 |
| 16 | Evaluate how CHWs can improve referral and counter-referral | 0.958 | 0.531 | 0.782 | 0.681 | 0.750 | 0.795 | 0.031 |
| 17 | Evaluate ways to assure continuous supply of essential medicines and inputs for CHWs | 0.975 | 0.558 | 0.718 | 0.612 | 0.847 | 0.791 | 0.111 |
| 18 | Evaluate the barriers at the community and provider level that cause poor referral | 0.975 | 0.521 | 0.741 | 0.741 | 0.793 | 0.789 | 0.042 |
| 19 | Evaluate ways to garner community support to ensure early and sustained breastfeeding | 0.921 | 0.696 | 0.800 | 0.686 | 0.781 | 0.775 | 0.111 |
| 20 | Evaluate ways to improve retention of CHWs | 0.967 | 0.529 | 0.786 | 0.703 | 0.892 | 0.768 | 0.135 |
| 21 | Evaluate how to measure good supervision for CHWs and different ways of providing it | 0.950 | 0.547 | 0.821 | 0.667 | 0.742 | 0.761 | 0.068 |
| 22 | Assess methods to ensure rational drug use among CHWs | 0.930 | 0.520 | 0.722 | 0.736 | 0.728 | 0.756 | 0.107 |
| 23 | Evaluate ways to maintain CHW neonatal resuscitation skills | 0.908 | 0.635 | 0.740 | 0.777 | 0.705 | 0.752 | 0.118 |
| 24 | Assess the optimal number of activities and population coverage required to maintain case load and skills of CHWs | 0.917 | 0.592 | 0.845 | 0.603 | 0.741 | 0.752 | 0.099 |
| 25 | Evaluate the equity impacts and effectiveness of CHW services when delivered with user fees or drug cost-recovery fees | 0.825 | 0.349 | 0.704 | 0.754 | 0.860 | 0.740 | 0.026 |
| 26 | Determine how CHWs can use injectable antibiotics for newborn sepsis safely and effectively | 0.800 | 0.824 | 0.764 | 0.789 | 0.708 | 0.739 | -0.111 |
| 27 | Determine how to adapt and improve existing diets for malnourished pregnant women based on home available foods | 0.925 | 0.500 | 0.588 | 0.691 | 0.704 | 0.737 | -0.057 |
| 28 | Evaluate different management structures for CHWs (community based, government based, private sector franchising, etc) | 0.956 | 0.402 | 0.798 | 0.661 | 0.769 | 0.732 | 0.080 |
| 29 | Determine culturally appropriate means to deliver skin to skin care (formative research of the cultural barriers, design of local solutions) | 0.900 | 0.570 | 0.745 | 0.821 | 0.830 | 0.724 | 0.060 |
| 30 | Assess the costs of individual interventions and combined packages of interventions | 0.930 | 0.363 | 0.685 | 0.673 | 0.714 | 0.721 | 0.149 |
| 31 | Determine how to overcome the cultural barriers for the adequate food intake of women during pregnancy in specific contexts like South Asia | 0.917 | 0.431 | 0.594 | 0.683 | 0.676 | 0.717 | 0.091 |
| 32 | Evaluate different ways of supporting facilitators of community engagement (training, supervision, skill maintenance, etc) | 0.921 | 0.451 | 0.836 | 0.527 | 0.684 | 0.706 | 0.091 |
| 33 | Evaluate different training approaches (including refresher training) for CHWs and their supervisors | 0.942 | 0.470 | 0.781 | 0.647 | 0.629 | 0.705 | 0.063 |
| 34 | Evaluate methods to prevent misuse of oxytocics | 0.875 | 0.444 | 0.660 | 0.717 | 0.708 | 0.705 | -0.073 |
| 35 | Evaluate methods to overcome health professional resistance to CHWs prescribing and administering drugs (e.g. oral or injectable antibiotics for newborn sepsis, injectable vitamin K, etc)? | 0.807 | 0.600 | 0.647 | 0.640 | 0.632 | 0.684 | 0.088 |
| 36 | Evaluate current career pathways for CHWs and methods for improving their prospects | 0.917 | 0.311 | 0.688 | 0.658 | 0.763 | 0.682 | 0.057 |
| 37 | Assess what communities consider as maternal-newborn health priorities and how communities compare maternal-newborn health with other development priorities | 0.877 | 0.290 | 0.627 | 0.447 | 0.772 | 0.672 | 0.100 |
| 38 | Evaluate various forms of community engagement (village health committees, mothers groups, working with religious leaders, community theatre and songs, etc) | 0.868 | 0.302 | 0.736 | 0.536 | 0.741 | 0.662 | 0.000 |
| 39 | Evaluate the different methods of selecting CHWs (community vs. competency based, etc) | 0.883 | 0.406 | 0.625 | 0.575 | 0.758 | 0.659 | -0.044 |
| 40 | Assess methods to ensure community awareness and practice of rational drug use | 0.868 | 0.388 | 0.673 | 0.585 | 0.759 | 0.659 | -0.033 |
| 41 | Assess the gender and other equity dimensions of community engagement | 0.813 | 0.330 | 0.690 | 0.670 | 0.589 | 0.656 | -0.058 |
| 42 | Measure the extent of household expenditures and their equity impacts | 0.891 | 0.226 | 0.433 | 0.613 | 0.704 | 0.656 | -0.018 |
| 43 | Evaluate different CHW labour arrangements (unionisation, levels of formalisation -volunteers vs. paid, etc) | 0.888 | 0.363 | 0.675 | 0.583 | 0.708 | 0.653 | 0.018 |
| 44 | Evaluate the impact on preventive and promotive aspects of community level interventions, when curative interventions are introduced into the package of services | 0.818 | 0.489 | 0.604 | 0.667 | 0.660 | 0.651 | 0.067 |
| 45 | Evaluate ways in which communities are involved in monitoring and evaluation | 0.895 | 0.367 | 0.704 | 0.509 | 0.763 | 0.647 | 0.099 |
| 46 | Assess the gender distribution of CHWs and its implications in terms of their acceptability and effectiveness | 0.925 | 0.343 | 0.602 | 0.619 | 0.633 | 0.639 | 0.114 |
| 47 | Assess how CHWs and other kinds of frontline health workers are represented in human resource policies, strategies and legislation | 0.925 | 0.271 | 0.556 | 0.583 | 0.692 | 0.638 | 0.092 |
| 48 | Evaluate methods of integrating community-based data collection into district HMIS | 0.930 | 0.298 | 0.636 | 0.526 | 0.579 | 0.628 | 0.131 |
| 49 | Evaluate methods and levels of accountability that can be ensured | 0.650 | 0.345 | 0.565 | 0.510 | 0.608 | 0.618 | 0.082 |
| 50 | Assess the methods of tracking budget allocations and flow | 0.889 | 0.256 | 0.482 | 0.636 | 0.651 | 0.611 | 0.062 |
| 51 | Determine the minimum set of indicators required and the most effective monitoring system | 0.825 | 0.298 | 0.609 | 0.535 | 0.544 | 0.608 | 0.082 |
| 52 | Evaluate the sequencing and linking of different community level interventions | 0.696 | 0.385 | 0.610 | 0.479 | 0.590 | 0.591 | 0.055 |
| 53 | Evaluate different stages of community engagement (consultation, cooperation, co-learning, collective action), including their phasing, cost and effectiveness | 0.816 | 0.267 | 0.663 | 0.453 | 0.548 | 0.587 | 0.145 |
| 54 | Evaluate ways to ensure delayed cord clamping in deliveries assisted by private providers | 0.933 | 0.278 | 0.478 | 0.343 | 0.616 | 0.573 | 0.147 |
| 55 | Assess the optimal number of community groups that a community engagement facilitor can support | 0.923 | 0.208 | 0.471 | 0.365 | 0.611 | 0.562 | 0.013 |
